# Multiple small hemorrhagic infarcts in cerebral air embolism: a case report

**DOI:** 10.1186/s13104-017-2925-x

**Published:** 2017-11-16

**Authors:** Masaya Togo, Taku Hoshi, Ryosuke Matsuoka, Yukihiro Imai, Nobuo Kohara

**Affiliations:** 1 Department of Neurology, Kobe City Medical Center General Hospital, Hyogo, Japan; 2 Department of Neurology, Kyoto University Graduate School of Medicine, Kyoto, Japan; 3 Department of Pathology, Kobe City Medical Center General Hospital, Hyogo, Japan

**Keywords:** Cerebral air embolism, Small hemorrhagic infarction, T2 star-weighted imaging

## Abstract

**Background:**

Cerebral air embolism is a rare cause of cerebral infarction. In cerebral air embolism, T2 star-weighted imaging shows numerous spotty hypointense signals. Previous reports have suggested that these signals represent air in the brain and are gradually diminished and absorbed. We experienced two cases of cerebral air embolism, and in one of them, we conducted an autopsy.

**Case presentation:**

Case 1 was a 76-year-old Japanese man with lung cancer and emphysema. A spasmodic cough induced massive cerebral and cardiac air embolisms and the patient died because of cerebral herniation. T2 star-weighted imaging of brain magnetic resonance imaging showed multiple spotty low signals. Brain autopsy showed numerous spotty hemorrhagic infarcts in the area of T2 star-weighted imaging signals. Case 2 was an 85-year-old Japanese man with emphysema who suffered from acute stroke. Similar spotty T2 star-weighted imaging signals were observed and remained unchanged 2 months after the onset.

**Conclusions:**

These findings indicate that T2 star-weighted imaging in cerebral air embolism partially represents micro-hemorrhagic infarction caused by air bubbles that have migrated into the brain.

## Background

Cerebral air embolism is a rare cause of cerebral infarction. Reported cases of cerebral air embolism are associated with trauma or iatrogenic accidents, such as intravascular catheterization [1]. In these cases, T2 star-weighted imaging (T2*WI) of brain magnetic resonance imaging (MRI) shows multiple spotty low-intensity signals in the infarcted area. These signals are thought to be air in the brain, and diminish soon after the onset of the air embolism [2].

We report two cases of air embolism based on lung disease. Both cases showed multiple spotty signals of T2*WI and one autopsy case showed spots representing hemorrhagic infarction, probably caused by micro air embolism. To the best of our knowledge, this is the first report of an autopsy showing multiple small hemorrhagic infarcts in cerebral air embolism.

## Case 1

The patient was a 76-year-old Japanese man with a history of a right lower lobectomy for lung adenocarcinoma. The patient also had a history idiopathic pulmonary fibrosis and emphysema, but did not have any history of amyloid angiopathy, and did not take anti-platelet drug or anticoagulants. He had a sudden onset of left hemiparesis and was admitted to our hospital. He had a blood pressure of 117/72 mmHg and a regular heart rate of 76 beats/min. He had disturbed consciousness, eye deviation to the right, and left hemiparesis. Diffusion-weighted magnetic resonance imaging (DWI) showed high-signal intensity areas in the right parietal lobe and T2*WI showed many hypointense spots in the same area.

**Fig. 1:**
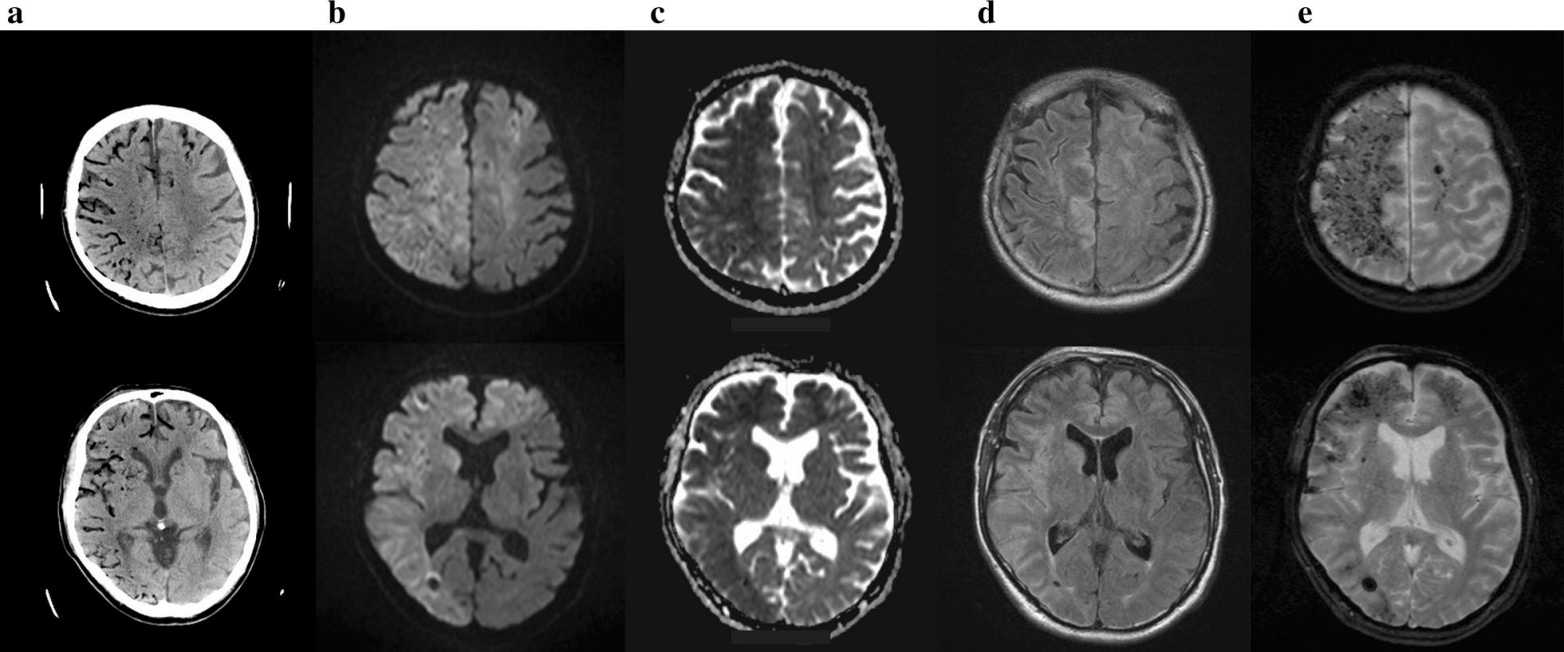
Brain CT and MRI of case 1. **a** Brain CT shows numerous foci of intravascular air within the area supplied by bilateral anterior cerebral arteries (ACAs) and the right middle cerebral artery (MCA). **b** Diffusion-weighted imaging (DWI) and **d** fluid attenuated inversion recovery (FLAIR) images show high-intensity areas that are supplied by the bilateral anterior cerebral arteries and the right middle cerebral artery. **c** Hypointense signal was observed on the apparent diffusion coefficient (ADC) map. **e** T2 star-weighted imaging (T2*WI) shows many hypointense spots in the area perfused by the bilateral anterior cerebral arteries and the right middle cerebral artery

**Fig. 2:**
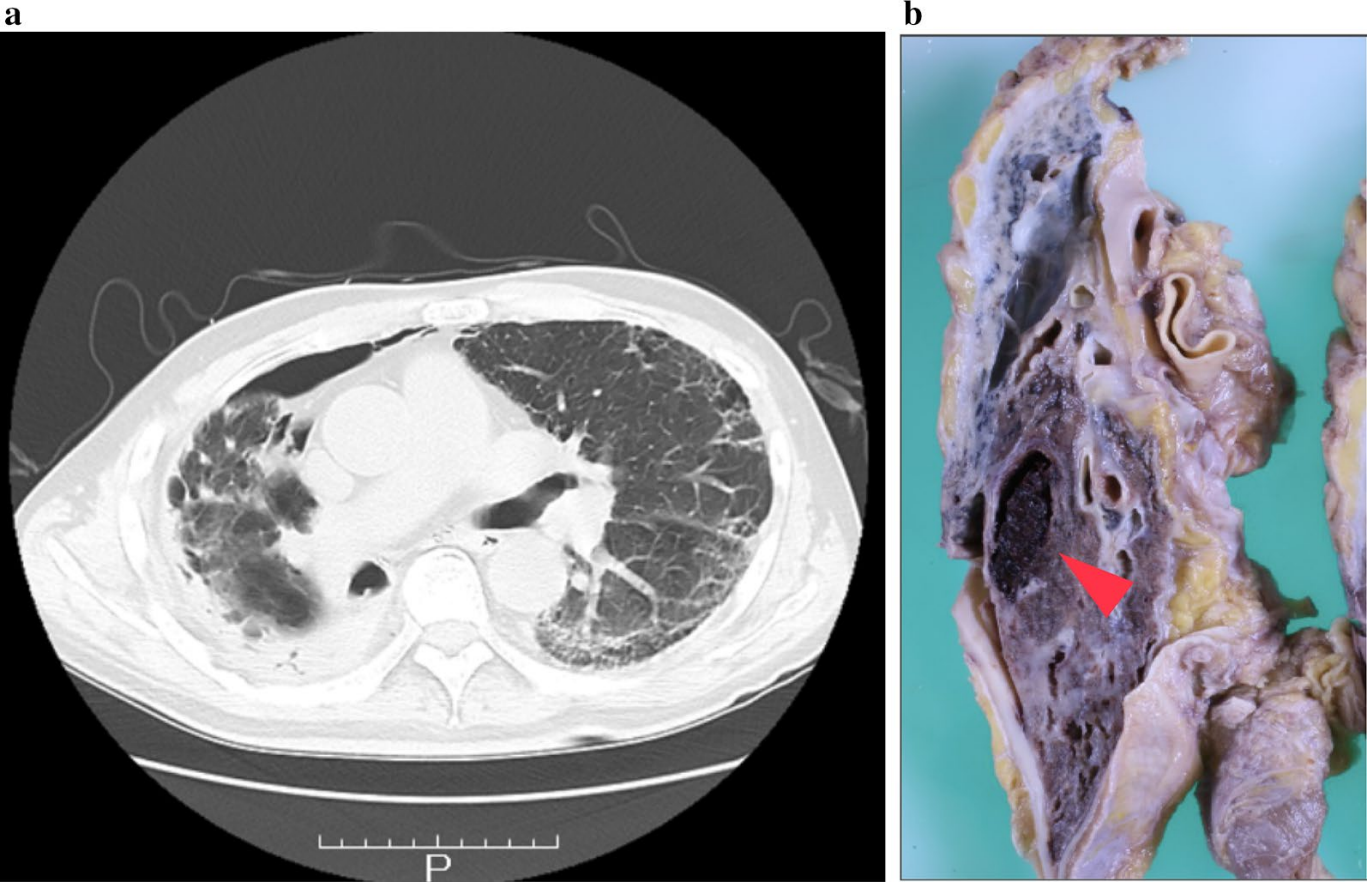
Chest CT and lung autopsy of case 1. **a** Chest CT shows bilateral emphysema and pneumothorax. **b** Lung autopsy shows many cysts and one cyst in the right middle lobe contains a relatively new hemorrhage (red arrowhead)

After admission, his symptoms gradually resolved. However, 12 h later, He was seated on his bed and he started violently coughing before sleeping in the evening. He kept coughing for 2 or 3 min. He coughed so violently that he could not speak with the nursing staff. During the violent coughing, he suddenly showed altered consciousness and developed a left hemiparesis. Emergent computed tomography (CT) revealed numerous foci of intravascular air within the area of the bilateral anterior cerebral arteries (ACA) and the right middle cerebral artery (MCA) (Fig. 1a). MRI showed high-signal intensity lesions on DWI (Fig. 1b) and fluid attenuated inversion recovery (FLAIR) imaging (Fig. 1d). The apparent diffusion coefficient (ADC) map showed hypointensity (Fig. 1c), which was consistent with the findings for acute ischemic stroke. In addition to this, there were many hypointense spots on T2*WI (Fig. 1e). Magnetic resonance angiography did not show obstruction or stenosis of intracranial blood vessels. Causes of stroke, such as cardiac embolism or dissection, other than air embolism were not likely because continuous ECG monitoring was used to both patients in the intensive care unit or wards, and body CT did not show the embolic source or dissection. Therefore, We diagnosed this patient as having a cerebral air embolism. His heart rate had dropped to 30 beats/min, and electrocardiography showed ST-segment elevation in leads II, III, and aVF, which suggested myocardial infarction. We carried out emergency coronary angiography and found no obstruction in the right coronary artery. After angiography, the ST-segment elevation in leads II, III, and aVF gradually improved. These findings were consistent with air embolization and reperfusion in the coronary artery. Although the differential diagnosis of air embolism included trauma or an invasive procedure, the patient did not have a history of trauma and did not undergo procedures, such as biopsy, placement of intravascular catheters, or mechanical ventilation, after admission. We could not find a patent foramen ovale by transthoracic and transesophageal echocardiography. Chest CT did not show pulmonary arteriovenous fistula or other sites communicating with blood vessels and air spaces, but it revealed severe emphysema and a pneumothorax (Fig. 2a). Because the patient’s symptoms occurred in the middle of coughing, we suspected that air migrated from the area of the emphysema or pneumothorax to the pulmonary vein and caused cerebral air embolism when he violently coughed.

**Fig. 3:**
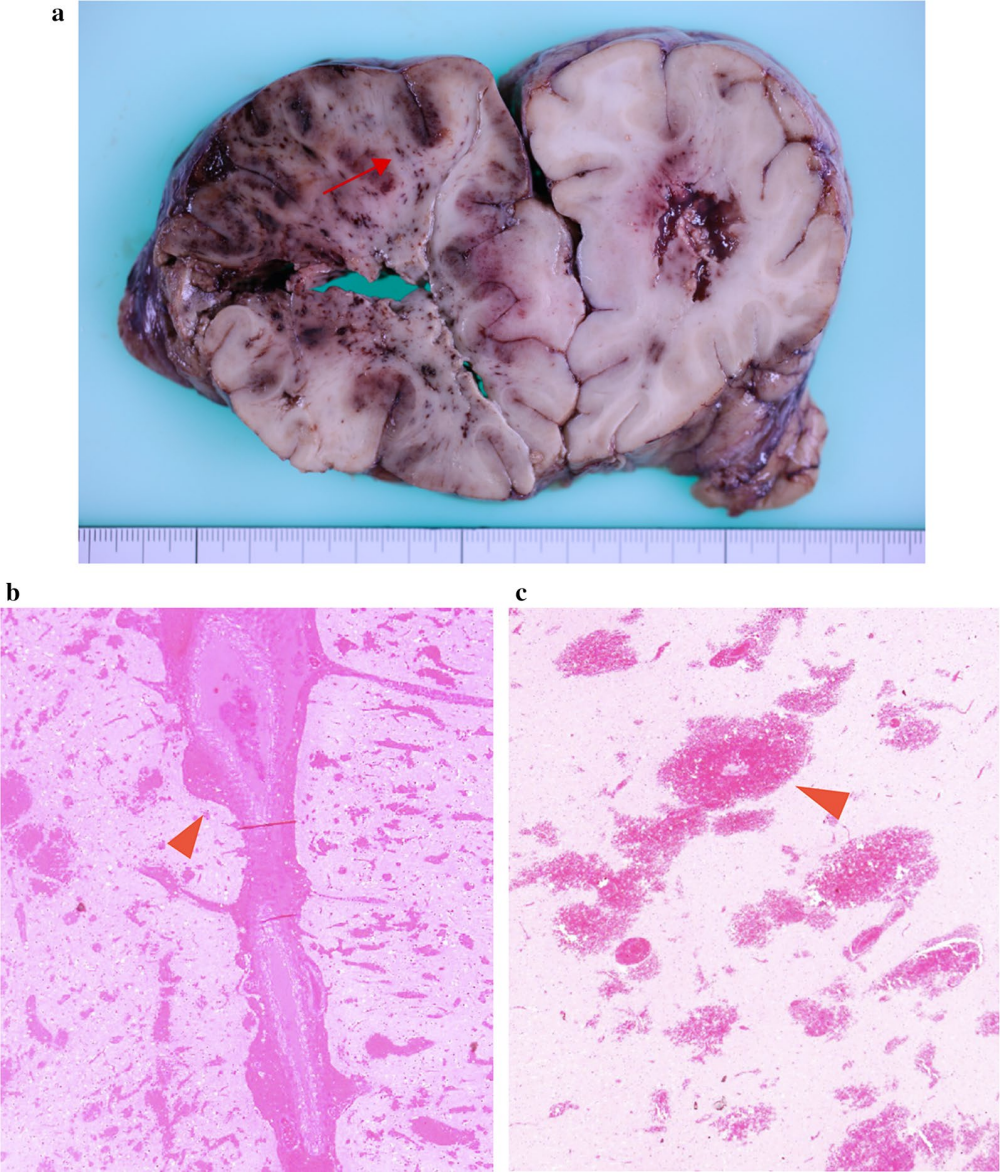
Brain autopsy of case 1. **a** There are many small hemorrhages in an infarcted area. **b** Microscopic examination of a small hemorrhage (red arrow in **a**) shows many hemorrhagic infarcts at low power (hematoxylin and eosin staining, ×20 magnification). **c** A high-power field of view (hematoxylin and eosin staining, ×100 magnification) by microscopic examination shows hemorrhagic infarcts around arterioles (arrowhead)

The next day, brain edema was aggravated, and we carried out external decompression. However, the brain edema worsened and the patient died on the third day of hospitalization because of brain herniation. We performed an autopsy and found myocardial hemorrhagic infarction in the left ventricle. His lungs were emphysematous with many cysts. One cyst in the right middle lobe contained relatively new hemorrhage (Fig. 2b, arrowhead), which suggested that the cyst communicated with vessels and could be an entry site for air. On autopsy of the brain, we found many small, hemorrhagic infarcts in the right cerebral hemisphere (Fig. 3a). Microscopic examination of the brain showed numerous hemorrhages (Fig. 3a, arrow). Hematoxylin and eosin staining of the cerebral white matter showed that hemorrhage occurred around the arterioles, at 20× magnification (Fig. 3b, arrowhead) and at 100× magnification (Fig. 3c, arrowhead). The pathological findings are suspected to be due to hemorrhages after cerebral air embolism and reperfusion.

## Case 2

**Fig. 4:**
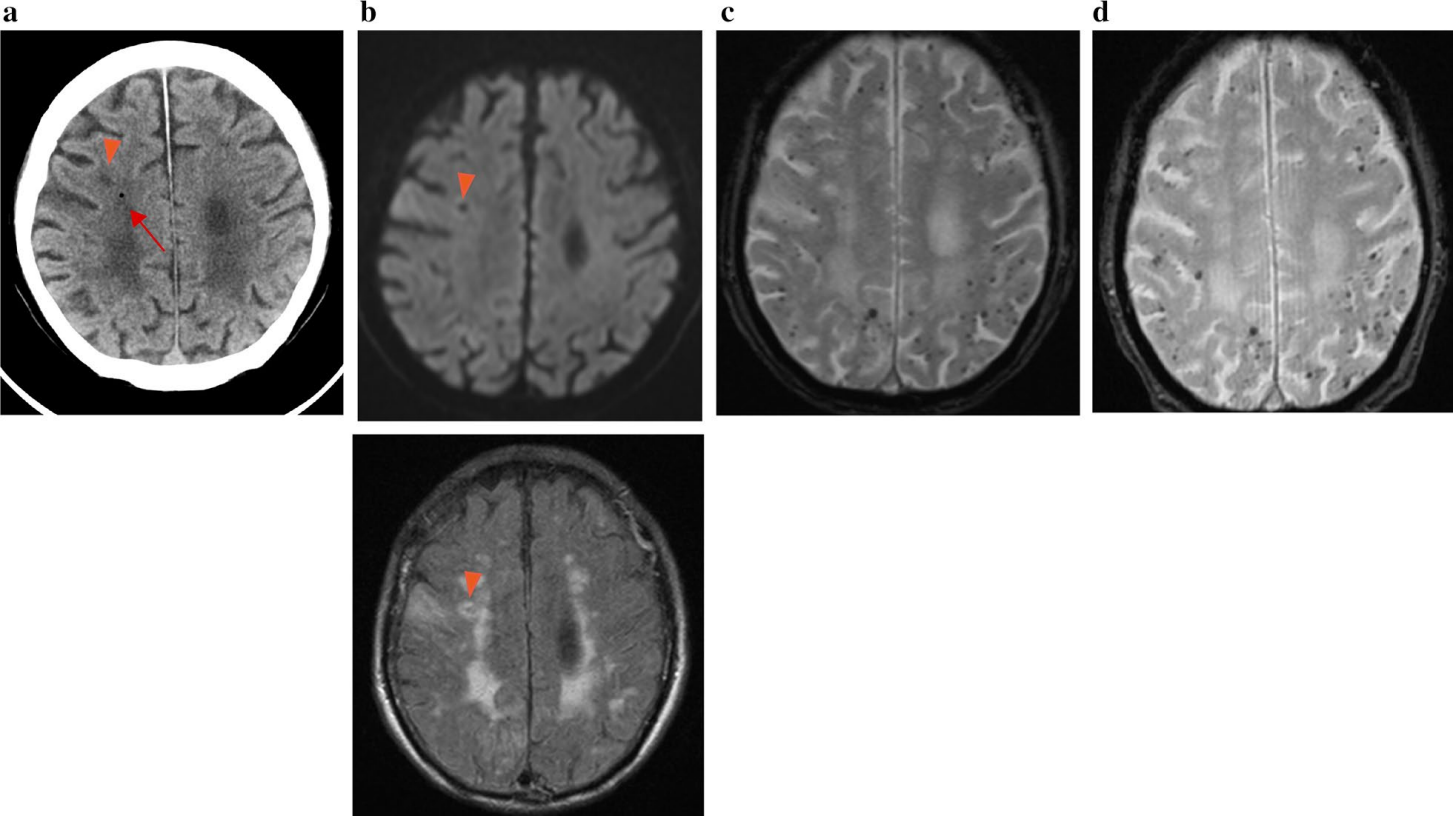
Brain CT and MRI of case 2. **a** Brain CT on admission shows a low-density spot in the right frontal lobe (red arrow) and old infarction (red arrowhead). **b** diffusion-weighted imaging (DWI) and fluid attenuated inversion recovery (FLAIR) images showed old infarction (red arrowhead), did not show obvious signals corresponding to the low-density spot on brain CT. **c** T2 star-weighted imaging (T2*WI) of MRI of case 2 on admission shows many hypointense signals in bilateral frontoparietal lobes. **d** T2 star-weighted imaging at 78 days after onset shows that the distribution of signals did not change compared with T2 star-weighted imaging on the admission day

The patient was an 85-year-old Japanese man with hypertension, bronchiectasis, and emphysema and did not have any history of amyloid angiopathy, did not take anti-platelet drug or anticoagulants. He presented to our hospital with left hemiparesis and disturbed consciousness. On arrival, his blood pressure was 169/89 mmHg with a regular heart rate of 76 beats/min. He showed mild disturbed consciousness and left hemiparesis. Brain CT did not show any abnormality on admission. After admission, his symptoms immediately resolved. At noon on the day of admission, the patient was seated on the bed and could eat lunch. However, after swallowing food, he showed altered consciousness and went into convulsions involving the left upper and lower extremities. A brain CT showed a low-density spot that was suspected to be air in the right frontal lobe (Fig. 4a, arrow). DWI and FLAIR images did not show obvious signals at the same location. The low-intensity spot in the right cerebral hemisphere corresponded to an old infarction. (Figure 4a, b, arrowhead). DWI and FLAIR images also showed a faint high-intensity signal and cortical swelling in the right dorsal part of the frontal lobe (not shown), but these changes were due to the acute symptomatic seizure that continued for approximately 30 min. Many hypointense lesions in the bilateral frontoparietal lobe were seen on T2*WI (Fig. 4c). The next day after onset of the events, the low-density spot on the brain CT disappeared, a change consistent with the findings of cerebral air embolism.

We diagnosed this patient as having cerebral air embolism and started to search for the embolic source. He did not have any history of trauma and did not undergo an invasive procedure after his admission. We did not detect a patent foramen ovale or any other right-to-left shunt in the whole body CT transthoracic echocardiography and micro-bubble transcranial Doppler. Several bullae and severe emphysema was seen on his chest CT images. He had difficulty in swallowing after admission and required a lot of effort to swallow food. It was therefore suspected that during swallowing, the intrathoracic pressure increased. Given this history and the chest CT findings, we concluded that air had migrated into the blood vessels from his lungs. After he was administered with an antiepileptic drug, he had no recurrence of seizures. His symptoms had not resolved (modified Rankin Scale 5) by the 28th days of hospitalization, and he was discharged from the hospital. On the 78th day after onset, he underwent brain MRI. Numerous hypointense, spotty signals remained on T2*WI. The distribution of these signals was similar to those on the day of admission (Fig. 4d). The findings suggest that the hypointense signals on T2*WI were not of air. Taking the autopsy results of case 1 into consideration, the signals could indicate small hemorrhages after air embolism.

## Discussion

Our two cases showed embolism in the brain after the events in which intrathoracic pressure was increased. Causes of stroke, such as cardiac embolism or dissection, other than air embolism were not likely because continuous ECG monitoring did not show atrial fibrillation in both patients, and body CT did not show the embolic source or dissection. Another study, transthoracic or transesophageal echocardiography, the micro-bubble test did not show the causes of stroke. Considering their clinical history, brain CT and MRI findings, we concluded air embolism was the cause of these two cases.

There are two categories of cerebral air embolism, venous and arterial. Venous cerebral air emboli result from air entering the jugular veins when a central venous catheter is removed. However, embolism can be arterial when the volume of the venous air exceeds the capacity of the pulmonary filter when there is right-to-left intra-cardiac shunting, and when air directly enters the systemic circulation, such as the pulmonary vein. On CT findings, serpiginous air density is compatible with venous air embolism, but arterial air embolism shows punctate low density in the brain parenchyma [2]. In both of our cases, air density showed a punctate pattern in the brain parenchyma, therefore we concluded that our cases were consistent with arterial air embolism. Although part of the air density in case 1 was serpiginous, when a lot of air enters the brain, CT sometimes shows air density along the artery and this density appears to be serpiginous [3].

Air embolism causes pathological changes by two mechanisms: mechanical obstruction and an inflammatory response to the bubble, resulting in cerebral edema and secondary ischemia [1]. Faint high-intensity DWI in our patients may reflect such inflammatory changes due to air embolism. Some reports have shown that the prognosis of cerebral air embolism is relatively good [4, 15, 21]. The reason for this finding may be because the amount of air is small and neurological dysfunction is not due to mechanical obstruction, but due to inflammatory change.

Cerebral air embolism usually occurs by traumatic or iatrogenic causes. Various causes have been reported, such as central venous catheter removal [1], scuba diving [4], CT-guided lung biopsy [5], endoscopic retrograde cholangiopancreatography [6], cerebral aneurysmal coiling [7], and pleural lavage [8]. As rare causes, pulmonary barotrauma or mechanical ventilation can also cause air embolism [9]. In our cases, patients have only intravenous line and such embolic causes, including invasive procedure, indwelling catheter and right-to-left shunt, did not present. Both of our patients had chronic pulmonary disease and cerebral air embolisms could have occurred when their intrathoracic pressure was increased, such as when coughing or swallowing occurred. Coughing has been reported to increase airway pressure and cause cerebral air embolism during lung biopsy. Additionally, if vessels are affected by inflammatory disease, a hemostatic mechanism might be impaired. This allows for continued patency of vessels and aids air embolism [5]. In other reports, authors have speculated that there are chronic inflammatory changes when patients have pulmonary disease, including a cavity created by aspergillosis infection [10], necrotic lung cancer [11], a tuberculosis cavity [12], or a pulmonary cyst [13]. In these situations, bronchovenous communication is formed. If there is an adequate pressure gradient, air can enter the systemic circulation through the bronchovenous communication and cause cerebral air embolism. We could not confirm bronchovenous communication at autopsy, but, in case 1, many pulmonary cysts were formed with pulmonary fibrosis. These cysts were suspected to be the entry site of the air, because bronchovenous communication could be formed in the cysts and air could enter into the blood vessels when intrathoracic pressure was increased. Our cases are rare because there was no traumatic or iatrogenic cause other than coughing or swallowing.

Treatment of cerebral air embolism is often supportive and hyperbaric therapy should be considered [15]. In our cases, although hyperbaric oxygen therapy was considered, the patients’ condition was too unstable to transfer the institution in which the therapy could be taken.

There have been 15 cases of air embolism with MRI findings [3–8, 11, 14–21]. Five of 15 cases showed multiple hypointense signals on T2*WI. Air has low magnetic susceptibility in the magnetic field, and it can cause a low-intensity signal on T2*WI [8, 19]. Moreover, these signals are thought to be air because many of them diminish over several days. With regard to brain pathological findings, reports of cerebral air embolism are rare. One report showed variably sized hemorrhagic infarctions [14], but it did not show small infarctions.

In our patients, case 1 showed many spotty hemorrhagic infarctions in the infarcted area. In case 2, the low-density area in brain CT did not match the hypointense signal on T2*WI, and many spotty signals remained 78 days after the onset. This finding is not consistent with the hypothesis that signals represent air in the brain. Part of the hypointense signal on T2*WI was likely to be air in the brain as previously reported [8]. However, as shown in this report, part of the hypointense signals represented multiple small hemorrhagic infarcts.

## Conclusions

Our cases of cerebral embolism are important in two aspects. First, cerebral air embolism could have been caused without traumatic or iatrogenic causes. Second, to the best of our knowledge, this is the first report showing that cerebral air embolism can cause multiple small hemorrhagic infarcts. Hypointense signals on T2*WI represent such small hemorrhagic infarcts.

## Abbreviations

ACA: anterior cerebral artery
ADC: apparent diffusion coefficient
CT: computed tomography
DWI: diffusion-weighted imaging
FLAIR: fluid attenuated inversion recovery
MCA: middle cerebral artery
MRI: magnetic resonance imaging
T2*WI: T2 star-weighted imaging
